# Prevalence and predictors of PTSD and low resilience symptoms among subscribers of the MoreGoodDays supportive text messaging program: A cross-sectional study

**DOI:** 10.1371/journal.pone.0339662

**Published:** 2026-01-09

**Authors:** Belinda Agyapong, Reham Shalaby, Ejemai Eboreime, Katherine Hay, Rachal Pattison, Mark Korthuis, Vincent Israel Opoku Agyapong

**Affiliations:** 1 Department of Psychiatry, Faculty of Medicine and Dentistry, University of Alberta, Edmonton, Alberta, Canada; 2 Department of Psychiatry, Faculty of Medicine, Dalhousie University, Halifax, Nova Scotia, Canada; 3 Kickstand, Edmonton, Alberta, Canada; 4 Glenrose Rehabilitation Hospital Foundation, Edmonton, Alberta, Canada; Kandahar University, Faculty of Medicine, AFGHANISTAN

## Abstract

**Background:**

Post-traumatic stress disorder (PTSD) is not uncommon among adolescents and young adults, with increased incidence after experiencing a traumatic event. MoreGoodDays, a self-subscription daily supportive text messaging program, co-designed with young adults, was launched in Alberta to provide free mental health support for adolescents and young adults to mitigate symptoms of stress, anxiety, depression, and PTSD.

**Objectives:**

This study aimed to determine the prevalence and predictors of probable PTSD and low resilience among subscribers of the MoreGoodDays supportive text messaging program.

**Methods:**

A cross-sectional online study was conducted among MoreGoodDays subscribers using the PTSD Checklist Civilian (PCL-C) version and the Brief Resilience Scale (BRS) scales. Data were analyzed with SPSS version 25. Chi-squared tests and multivariate logistic regression analysis were applied to identify predictors.

**Results:**

Of the 1,045, subscribers of MoreGoodDays program, 343 completed the survey. 45.7% of the respondents experience probable PTSD and 51.7% had low resilience. Receiving mental health counseling, desire for counseling, and high ACE scores were strong predictors of both outcomes. Participants who had received mental health counseling in the past year were 13.7 times more likely to experience PTSD symptoms (OR = 13.70; 95% CI: 1.23–142.86) and 15.15 times more likely to experience low resilience than those who did not (OR = 15.15; 95% CI: 1.46–166.67). Again, those who desired to receive mental health counseling were 20.8 times more likely to experience PTSD symptoms (OR = 20.76; 95% CI: 2.61–165.401) and 29.4 times more likely to experience low resilience than those who did not (OR = 29.42; 95% CI: 3.31–261.445). Finally, those with a score of four or more on the Adverse Childhood Experience Questionnaire were 6.2 times more likely to experience probable PTSD symptoms than participants with zero scores (OR = 6.24; 95% CI: 1.46–26.67).

**Conclusion:**

Probable PTSD and low resilience are prevalent, particularly among the youth with high ACE scores and those seeking mental health counseling, highlighting the need for targeted mental health promotion strategies. Government agencies and policymakers in the educational field for instance should endeavor to promote mental well-being by integrating mental health education into school curricula to build awareness and resilience.

## Introduction

Post-traumatic stress disorder (PTSD) is a common phenomenon among adolescents and young adults, with increased occurrence after physical abuse or other interpersonal trauma [1]. Research shows that a large proportion of adolescents (61.8%) experience a traumatic event in their lifetime, thus increasing their risk of PTSD [2]. The risk factors for PTSD in adolescents and young adults are comparable to the general population and include increased exposure to and perception of threat to self and other socio-demographic factors like age, gender, and educational level [3,4].

PTSD can also lead to role impairment, adverse emotional and psychological consequences, and increased risk of other mental and physical disorders [5]. Adolescents and young adults who experience PTSD also tend to suffer from emotion regulation difficulties, which are considered as features connected to but not core to PTSD symptoms [6]. One study reported that adolescents with PTSD experience increased levels of anxiety and depression, have difficulty coping at home and school, and tend to be involved in self-harm behaviors [7].

Ongoing adverse life stressors have generally been reported to be a risk factor for developing psychopathology after traumatic life events, and this is evident in the decline of resilience among individuals after cumulative stressors [4]. The number of traumatic experiences has also been reported to correlate with the prevalence and severity of the mental health disorders experienced [8]. For example, individuals who experienced the COVID-19 pandemic and another traumatic event, were ten times more likely to experience low resilience and PTSD than those who encountered trauma from only the COVID-19 pandemic [8]. This highlights the compounded effect of multiple traumatic events on mental health and underscores the need for targeted interventions for individuals experiencing trauma. A meta-analysis also reported a high prevalence of PTSD among college students during the pandemic [9], further highlighting the psychological impact of COVID-19 pandemic on the mental health of adolescents and young adults.

Psychological resilience is an individual’s ability to adapt, overcome stress, challenging life circumstances or adversity, and achieve positive outcomes [10]. High resilience has also been linked to a lesser tendency to experience anxiety, depression, and PTSD [11]. Further, resilient individuals usually have the capacity to navigate through crises, manage their emotions, and continue to function effectively even in the face of significant setbacks [12,13].

Previous studies in Alberta [14] using the Text4Hope program, focused primarily on the general population creating a critical gap in understanding the mental health burden and prevalence of psychological distress within the adolescents and young adult groups. The current study addresses this gap by focusing explicitly on the prevalence of probable PTSD, among adolescents and young adults in Alberta through the MoreGoodDays program.

MoreGoodDays, is a self-subscription daily supportive text messaging program, launched to provide free mental health support for young adults in Alberta. Specifically, MoreGoodDays [15,16] was co-designed with adolescents and young adults for their peers to mitigate symptoms of stress, anxiety, depression, and PTSD and improve resilience during the COVID-19 pandemic and beyond. It is one of the ResilienceNHope suite of evidence-based text and email messaging programs offered to the general population before, during, and after the pandemic in Alberta to support their mental health [17–20]. Moreover, evidence-based programs such as Text4Hope demonstrate the effectiveness of supportive text-messaging interventions for youth mental health [21]. These findings further contextualize the effectiveness of the MoreGoodDays program in supporting the mental well-being of the youth. With the impact of the COVID-19 pandemic and other stressors, it was vital to explore and ascertain the impact of the pandemic on adolescents and young adults. Also, knowledge about the prevalence and predictors of probable PTSD and low resilience in this cohort can have significant implications for mental health and public health strategies to address these problems. Furthermore, results from this study could inform targeted, scalable mental health interventions or policy decisions for young adults and youth populations. In this light, the objective of this study is to examine the prevalence and predictors of probable PTSD and low resilience among MoreGoodDays subscribers.

## Methodology

### Study settings and design

This study employed a cross-sectional study design. As part of the MoreGoodDays program, subscribers (participants) were invited to complete an online survey questionnaire completed upon enrolment to the service. Adolescents and young adults with a minimum age of 18 years and less than or equal to 26 years in Alberta could subscribe to the MoreGoodDays program by texting ‘MoreGoodDays’ to a designated short code number to receive free daily text messages for six months [15]. For the purpose of accessing age-related addiction and mental health services in Alberta, Canada, young adults have been categorized as between 17 and 26 years [22].

### Ethics

The University of Alberta Health Research Ethics Committee (Pro00106957) granted approval for the study) on January 5^th^, 2021. The ethics board approved implied consent when participants complete and return the online survey. The studies were conducted in accordance with the local legislation and institutional requirements. The ethics committee/institutional review board waived the requirement of written informed consent for participation from the participants or the participants’ legal guardians/next of kin because this is an anonymous online survey and so written informed consent will not have been possible. Participants had access to the information leaflet accompanying the survey questions at the beginning of the online survey, notifying them that consent was implied upon completion and submission of the online survey. Thus, informed consent was obtained from all subjects. Respondents’ privacy and data protection were assured throughout the study as no participants identifiable data was collected. Only data from individuals 18 years and older are reported in this study.

### Data collection

Data collection commenced from January 28, 2021, to July 17, 2022, using an anonymous online survey delivered to subscribers of the MoreGoodDays program. Participants self-subscribed to the MoreGoodDays program by texting the word MoreGoodDays to the designated number for the program so they receive daily supportive messages [23]. Subscribers could also text “STOP” anytime to unsubscribe from the MoreGoodDays program. The completion of the survey usually takes approximately 10 minutes. Each subscriber receives the same message daily during their subscription period.

All adolescents and young adults in Alberta could subscribe to the program and were invited through a link that accompanied the initial text message to complete the survey. However, access to mobile device was required in order to subscribe to MoreGoodDays program, thus excluding individuals without mobile phones. Completing the survey was independent of receiving text messages. Kickstand Edmonton was responsible for the promotion of the program [24]. The online survey, which was embedded in the welcome or initial text message received by subscribers of MoreGoodDays, included a blend of questions assessing categorical socio-demographic and clinical variables, including age, gender, ethnicity, educational level, marital status, adverse childhood experience, history of mental health conditions, willingness to/receipt of counseling. The baseline demographic questions, though not validated, have been used in previous studies by authors [24].

### Outcome measures

Probable PTSD and resilience level among study subscribers were the primary outcome measures of this study and were evaluated using the PTSD Checklist Civilian (PCL-C) (PCL-C score of 44 or more for probable PTSD) [25] and the Brief Resilience Scale (BRS) (a mean score ≤2.99 on the BRS indicates low resilience) [26]. The scales were studied as categorical variables for the purpose of prevalence estimates. For the severity estimates, we used the total scores for PCL-C (responses range from 17 to 85, and lower scores denote the better condition) and BRS (responses range from 6 to 30, and higher scores denote the better condition). The PCL-C test scores demonstrated good internal consistency (α = 0.96), test-retest reliability (r = 0.84), and convergent and discriminant validity [27]. The PCL-C scale was used to assess probable PTSD to allow comparability of prevalence estimates with those of a previous study in the Alberta population, which utilized the same scale [8, 28].

The BRS has good criterion validity and internal consistency (α = 0.71), with effectively established measures of well-being, optimism, self-esteem, self-efficacy, and psychological health [29]. These scales, although validated scales are not intended as diagnostic tools. Scores on the 10-item Adverse Childhood Experience (ACE-10) Questionnaire were included as predictors of probable PTSD and low resilience. The ACE-10 item is a self-rated questionnaire with items scoring YES or NO and is used to assess traumatic events during childhood [30, 31]. For the purpose of analysis, the ACE scores were grouped into five categories (0, 1, 2, 3, and ≥ 4) and included as predictor variables in the regression model [31].

### Sample size estimation

With 1,045 active subscribers to the MoreGoodDays service by July 17, 2022, a 95% confidence interval, and a ± 3% margin of error, the required sample size for prevalence estimates for probable PTSD or low resilience among MoreGoodDays program subscribers will be 529 [32]. The same calculation adopted in the estimation of prevalence of mental health conditions in subscribers of related supportive text messaging programs, was utilized in this study for consistency and effective comparison [8, 24].

### Statistical analysis

SPSS Version 25 (IBM Corp 2011) [33] was used for the data analysis. Descriptive statistics were applied for demographic and clinical data based on the age of the respondents. Chi-squared analysis was utilized to evaluate all the variables in relation to the probable PTSD present or absent and resilience categorical variable (low and moderate-to-high resilience). Two binary logistic regression models were used to find the significant predictors of probable PTSD and low resilience. The model included the significant (p < 0.05) and near significant (0.1 ≥ p ≥ 0.05) Variables with the probable PTSD or low resilience obtained from the univariate analysis. Odds ratios (OR) and confidence intervals (CI) were reported, determining the predictor variables for self-reported probable PTSD and low resilience while controlling for other variables in the model.

Correlational analysis was performed prior to the regression analysis to exclude any strong intercorrelations (Spearman’s correlation coefficient of 0.7 to 1.0 or − 0.7 to − 1.0) among potential predictors. We deleted participants who had any missing data on certain variables. Thus, there was no imputation of missing data and the reported data represented the complete responses. Duplicated surveys easily identified by participants phone numbers were also deleted.

## Results

**Fig 1:**
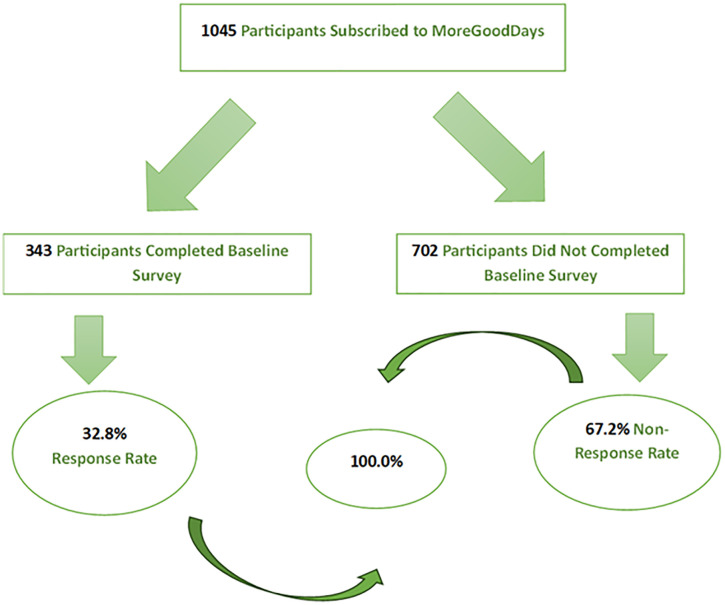
Study Flow Diagram.

**Fig 2:**
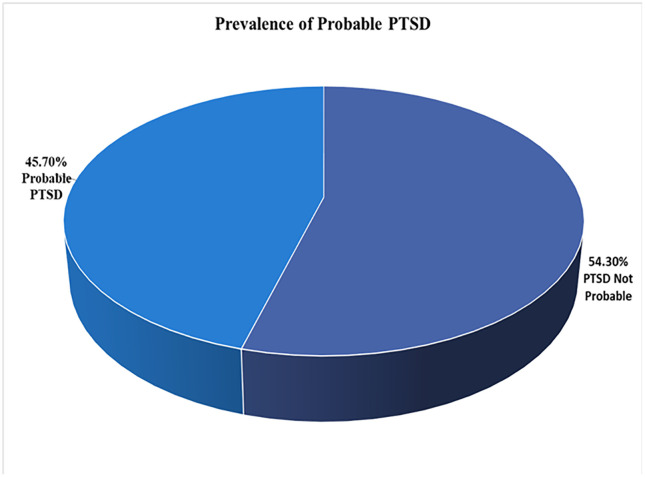
Prevalence of probable PTSD.

**Fig 3:**
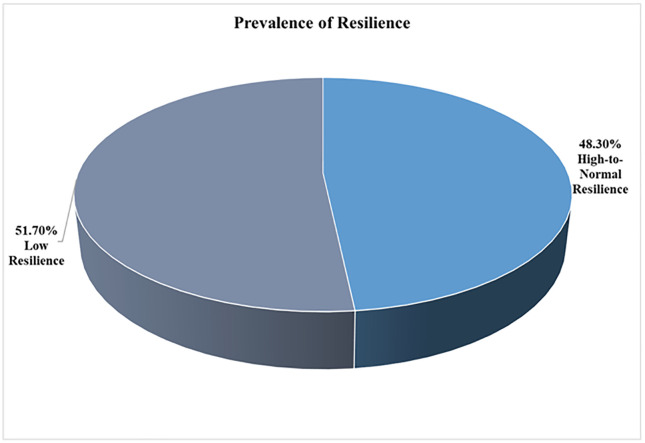
Prevalence of resilience.

**Fig 4:**
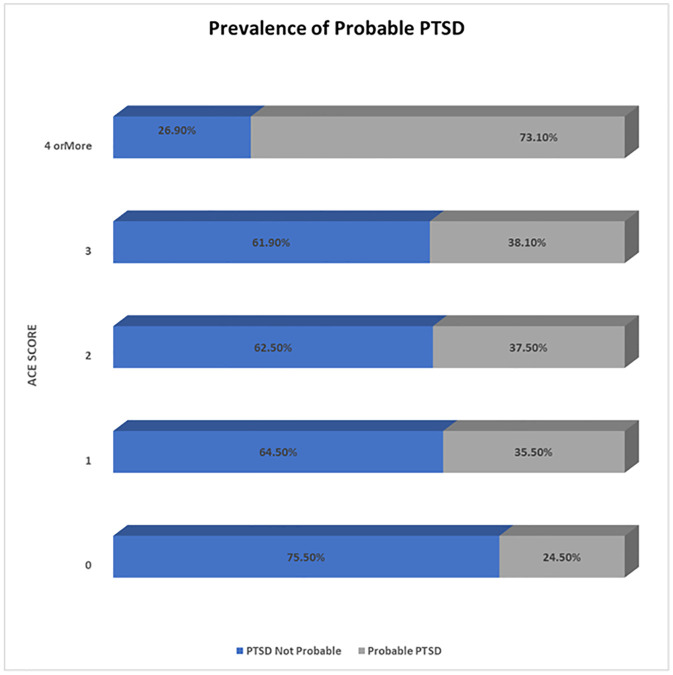
Prevalence of probable PTSD in relation to ACE Scores.

Overall, 343 out of 1,045 subscribers of MoreGoodDays participated in the baseline survey, representing a response rate of 32.8%, shown in Fig 1, the study flow diagram. An overview of the socio-demographic and clinical characteristics of the participants is shown in Table 1, Fig 2, Fig 3, and Fig 4. Most of the participants (250, 73.1%) were white, (163, 47.4%) employed, (82, 23.8%) students and lived with family and friends (148, 43.0%). Overall, 95 (45.7%) of respondents had probable PTSD, and 109 (51.7%) had low resilience. Additionally, 176 (51.3%) had received mental health counseling, and one-third desired to receive mental health counseling. Finally, the prevalence of probable PTSD among all the respondents was 45.7%, and the prevalence of low resilience among our respondents was 51.7%.

**Table 1:** Demographic and mental health-related variables distributed by age.

Variables	≤ 26 years n (%) N = 182	>26 years n (%) N = 162	Total n (%) N = 343
**Socio-demographic characteristics**			
**Gender**			
Male	24 (13.2%)	23 (14.3%)	47 (13.7%)
Female	136 (74.7%)	135 (83.9%)	271 (79.0%)
Other	22 (12.1%)	3 (1.9%)	25 (7.3%)
**Ethnicity**			
White	114 (63.0%)	136 (84.5%)	250 (73.1%)
Aboriginal	23 (12.7%)	14 (8.7%)	37 (10.8%)
Asian	28 (15.5%)	7 (4.3%)	35 (10.2%)
Other	16 (8.8%)	4 (2.5%)	20 (5.8%)
**Educational level**			
Less than high school	43 (23.5%)	7 (4.3%)	50 (14.5%)
High school	65 (35.5%)	10 (6.2%)	75 (21.8%)
Post-secondary education	75 (41.0%)	144 (89.4%)	219 (63.7%)
**Relationship status**			
In a relationship (married, common law, partnered)	43 (23.5%)	112 (69.6%)	155 (45.1%)
Single	139 (76.0%)	31 (19.3%)	170 (49.4%)
Separated/Divorced/Widowed	1 (0.5%)	18 (11.2%)	19 (5.5%)
**Employment status**			
Employed	41 (22.4%)	122 (75.8%)	163 (47.4%)
Unemployed	16 (8.7%)	23 (14.3%)	39 (11.3%)
Student	77 (42.1%)	5 (3.1%)	82 (23.8%)
Student and employed	49 (26.8%)	11 (6.8%)	60 (17.4%)
**Housing status**			
Own home	5 (2.7%)	101 (62.7%)	106 (30.8%)
Rented accommodation.	39 (21.3%)	51 (31.7%)	90 (26.2%)
Live with family or friends.	139 (76.0%)	9 (5.6%)	148 (43.0%)
**MH history**			
**Depression**			
No	103 (56.3%)	117 (72.7%)	220 (64.0%)
Yes	80 (43.7%)	44 (27.3%)	124 (36.0%)
**BD**			
No	179 (97.8%)	155 (96.3%)	334 (97.1%)
Yes	4 (2.2%)	6 (3.7%)	10 (2.9%)
**GAD**			
No	90 (49.2%)	119 (73.9%)	209 (60.8%)
Yes	93 (50.8%)	42 (26.1%)	135 (39.2%)
**Eating disorder**			
No	167 (91.3%)	150 (93.2%)	317 (92.2%)
Yes	16 (8.7%)	11 (6.8%)	27 (7.8%)
**OCD**			
No	166 (90.7%)	149 (92.5%)	315 (91.6%)
Yes	17 (9.3%)	12 (7.5%)	29 (8.4%)
**SUD**			
No	181 (98.9%)	155 (96.3%)	336 (97.7%)
Yes	2 (1.1%)	6 (3.7%)	8 (2.3%)
**Schizophrenia**			
No	182 (99.5%)	160 (99.4%)	342 (99.4%)
Yes	1 (0.5%)	1 (0.6%)	2 (0.6%)
**PD**			
No	179 (97.8%)	154 (95.7%)	333 (96.8%)
Yes	4 (2.2%)	7 (4.3%)	11 (3.2%)
**ADHD**			
No	172 (94.0%)	152 (94.4%)	324 (94.2%)
Yes	11 (6.0%)	9 (5.6%)	20 (5.8%)
**PTSD**			
No	156 (85.2%)	138 (85.7%)	294 (85.5%)
Yes	27 (14.8%)	23 (14.3%)	50 (14.5%)
**No MH history**			
No	71 (38.8%)	83 (51.6%)	154 (44.8%)
Yes	112 (61.2%)	78 (48.4%)	190 (55.2%)
**Medication Hx**			
**Antidepressants**			
No	124 (67.8%)	113 (70.2%)	237 (68.9%)
Yes	59 (32.2%)	48 (29.8%)	107 (31.1%)
**Antipsychotic**			
No	180 (98.4%)	154 (95.7%)	334 (97.1%)
Yes	3 (1.6%)	7 (4.3%)	10 (2.9%)
**Benzodiazepines**			
No	179 (97.8%)	153 (95.0%)	332 (96.5%)
Yes	4 (2.2%)	8 (5.0%)	12 (3.5%)
**Mood stabilizers**			
No	176 (96.2%)	154 (95.7%)	330 (95.9%)
Yes	7 (3.8%)	7 (4.3%)	14 (4.1%)
**Sleeping tablets**			
No	171 (93.4%)	150 (93.2%)	321 (93.3%)
Yes	12 (6.6%)	11 (6.8%)	23 (6.7%)
**Stimulants**			
No	177 (96.7%)	153 (95.0%)	330 (95.9%)
Yes	6 (3.3%)	8 (5.0%)	14 (4.1%)
**No medications**			
No	71 (38.8%)	59 (36.6%)	130 (37.8%)
Yes	130 (37.8%)	102 (63.4%)	214 (62.2%)
**Have you received MH counseling?**			
No	77 (42.1%)	90 (56.3%)	167 (48.7%)
Yes	106 (57.9%)	70 (43.8%)	176 (51.3%)
**Would you like to receive MH counseling**			
No	18 (20.5%)	39 (41.9%)	57 (31.5%)
Yes	42 (47.7%)	22 (23.7%)	64 (35.4%)
Unsure/Undecided	28 (31.8%)	32 (34.4%)	60 (33.1%)
**Clinical characteristics (scale used)**			
**ACE**			
**0**	28 (23.5%)	26 (26.3%)	54 (24.8%)
**1**	22 (18.5%)	12 (12.1%)	34 (15.6%)
**2**	17 (14.3%)	17 (17.2%)	34 (15.6%)
**3**	13 (10.9%)	11 (11.1%)	24 (11.0%)
**Four or more**	39 (32.8%)	33 (33.3%)	72 (33.0%)
**Resilience (BRS)**			
High-to-normal resilience	41 (36.3%)	61 (62.2%)	102 (48.3%)
Low resilience	72 (63.7%)	37 (37.8%)	109 (51.7%)
**PCL-C** Unlikely PTSD	46 (41.4%)	67 (69.1%)	113 (54.3%)
Probable PTSD	65 (58.6%)	30 (30.9%)	95 (45.7%)

**Abbreviations: BRS**: Brief Resilience Scale; **ACE:** Adverse Childhood Experiences; **MH:** Mental Health; **GAD:** Generalized anxiety Disorder; **PTSD:** Post Traumatic Stress Disorder; **Hx:** History; **PD:** Personality Disorder; **OCD:** obsessive-compulsive disorder; **SUD:** substance use disorder; BD borderline disorder; **ADHD:** attention deficit hyperactivity disorder. **PCL-C:** PTSD Checklist Civilian; * Fisher Exact Value; **df:** Degree of freedom; *Phi Value

Table 2 and Table in S1 Table shows that 17 variables had a significant association with likely Post Traumatic Stress Disorder (PTSD), including age, educational levels, relationship status, employment status, housing status, mental health (MH) history of depression, anxiety disorder, personality disorder (PD), attention deficit hyperactivity disorder (ADHD), PTSD, no mental health history, medication history of antidepressants, sleeping tablets, no medication history, have received MH counseling, and desire to receive mental health counseling. Participants aged 26 years or less, of ‘other’ gender, aboriginal, having a high school education, are students and employed, separated/divorced/widowed, and those renting accommodation had a higher prevalence of probable PTSD compared to other participants in their respective categories. Similarly, participants with a mental health history of depression, GAD, PD, ADHD, and PTSD, those with no mental health history, those on no medication or antidepressants, or sleeping tablets, those who would like to receive mental health counseling, or have received mental health counseling, and those with Adverse Childhood Experience (ACE) scores four or above had probable PTSD compared with other respondents in their respective categories.

**Table 2:** Chi-square test of association between demographic, mental health characteristics, and probable PTSD.

Variables	Unlikely PTSD n (%) N = 113	Probable PTSD n (%) N = 95	P value
**Socio-demographic characteristics**			
**Age (Years)**			< 0.001
≤26	46 (41.4%)	65 (58.6%)	
>26	67 (69.1%)	30 (30.9%)	
**Educational level**			< 0.001
Less than high school	9 (36.0%)	16 (64.0%)	
High school	16 (34.8%)	30 (65.2%)	
Post-secondary education	88 (64.2%)	49 (35.8%)	
**Relationship status**			* < 0.001
In a relationship (married, common law, partnered)	68 (71.6%)	27 (28.4%)	
Single	45 (43.3%)	59 (56.7%)	
Separated/Divorced/Widowed	0 (0.0%	9 (100.0%)	
**Employment status**			0.01
Employed	68 (64.8%)	37 (35.2%)	
Unemployed	14 (56.0%)	11 (44.0%)	
Student Student and employed	19 (41.3%) 12 (37.5%)	27 (58.7%) 20 (62.5%)	
		
**Housing status**			< 0.001
Own home	50 (80.6%)	12 (19.4%)	
Rented accommodation	22 (39.3%)	34 (60.7%)	
Live with family or friend	41 (45.6%)	49 (54.4%)	
**MH history**			
Depression			< 0.001
No	84 (66.1%)	43 (33.9%)	
Yes	29 (35.8%)	52 (64.2%)	
GAD			0.001
No	79 (64.2%)	44 (35.8%)	
Yes	34 (40.0%)	51 (60.0%)	
PD			*0.05
No	112 (55.7%)	89 (44.3%)	
Yes	1 (14.3%)	6 (85.7%)	
ADHD			0.04
No	110 (56.1%)	86 (43.9%)	
Yes	3 (25.0%)	9 (75.0%)	
PTSD			0.01
No	103 (58.2%)	74 (41.8%)	
Yes	10 (32.3%)	21 (67.7%)	
No MH history			< 0.001
No	64 (70.3%)	27 (29.7%)	
Yes	49 (41.9%)	68 (58.1%)	
**Medication Hx**			
Antidepressants			0.01
No	87 (60.0%)	58 (40.0%)	
Yes	26 (41.3%)	37 (58.7%)	
Sleeping tablets			< 0.001
No	112 (57.4%)	83 (42.6%)	
Yes	1 (7.7%)	12 (92.3%)	
No medications			0.04
No	33 (44.6%)	41 (55.4%)	
Yes	80 (59.7%)	54 (40.3%)	
**Have you received MH counseling?**			0.01
No	64 (63.4%)	37 (36.6%)	
Yes	49 (45.8%)	58 (54.2%)	
**Would you like to receive MH counseling**			0.001
No	27 (90.0%)	3 (10.0%)	
Yes	19 (47.5%)	21 (52.5%)	
Unsure/Undecided	25 (58.1%)	18 (41.9%)	
**ACE score**			< 0.001
**0**	40 (75.5%)	13 (24.5%)	
**1**	20 (64.5%)	11 (35.5%)	
**2**	20 (62.5%)	12 (37.5%)	
**3**	13 (61.9%)	8 (38.1%)	
**Four or more**	18 (26.9%)	49 (73.1%)	

*Fishers Exact Value.

Table 3 gives the logistic regression model for PTSD. Overall, 14 predictor variables that had a significant association or a trend towards significant association (*p* ≤ 0.05) or near significant association (0.1 ≥ *p* > 0.05) with probable PTSD on Chi-Square/ Fisher’s exact test were included in a binary logistic regression model. Three variables were excluded from the regression analysis since they were highly correlated (r ≥ 0.7) with other variables on correlation analysis. The excluded variables included “housing status,” “have never received mental health diagnosis,” and “not on any medication.” These variables were respectively highly correlated with “age,” “history of anxiety disorder, and “on antidepressants.” The rationale for exclusion in each case was that the included variables were thought to be of more relevance to the outcome of interest. The logistic regression model was statistically significant; *Χ*^2^ (df = 22; *n* = 351) = 58.88, *p* ≤ 0.001, indicating that the model could differentiate between study respondents with the presence and absence of probable PTSD. The model explained 41.2% (Cox and Snell R^2^) to 56.0% (Nagelkerke R^2^) of the variance and correctly classified 84.7% of cases. Three variables*, “Have you received mental health counseling in the past year?”* “*Would you like to receive mental health counseling and “ACE scores”* independently predicted the presence of probable PTSD in the respondents. When all other variables are controlled in the regression model, respondents who have received mental health counseling in the past year were 13.7 times more likely to experience PTSD symptoms than those who had not (OR = 13.70; 95% CI: 1.23–142.86). Again, those who desired to receive mental health counseling were 20.8 times more likely to experience PTSD symptoms than respondents who did not (OR = 20.76; 95% CI: 2.61–165.401). Similarly, those who were not sure if they would like to receive mental health counseling were 16.7 times more likely to experience PTSD symptoms than those who did not want to receive mental health counseling (OR = 16.67; 95% CI: 1.99–140.06). Finally, those who had four or more ACE scores were 6.2 times more likely to experience PTSD symptoms than those who had zero scores (OR = 6.24; 95% CI: 1.46–26.67).

**Table 3:** Output of the logistic regression model illustrating predictors of probable PTSD.

	Exp(B) Odd Ratio (OR)	Sig.	95% C.I.for EXP(B)	
			Lower	Upper
**Age ≤ 26 years**	0.612	0.574	0.110	3.392
**Education**				
Less than high school		0.227		
High school	0.177	0.118	0.020	1.555
Post-secondary education	0.162	0.102	0.018	1.436
**Relationship status**				
In a relationship (married, common law, partnered)		0.726		
Single	1.725	0.424	0.453	6.568
Separated/Divorced/Widowed	1459589183.274	0.999	0.000	
**Employment status**				
Employed		0.273		
Unemployed	3.211	0.198	0.543	18.986
Student	4.297	0.176	0.520	35.482
Student and employed	6.640	0.084	0.774	56.932
**Depression Disorder diagnosis? (Yes)**	2.507	0.253	0.519	12.111
**Anxiety disorder mental health diagnosis (Yes)**	2.210	0.350	0.419	11.645
**Have you received a Personality Disorder Diagnosis (Yes)**	35334120.235	1.000	0.000	
**ADHD diagnosis (Yes)**	3.105	0.524	0.095	101.548
**Have you received a PTSD or trauma-related disorder Diagnosis? (Yes)**	1.324	0.821	0.115	15.200
**Medication**				
**On Antidepressants, e.g., Prozac (Yes)**	0.426	0.415	0.055	3.319
**On Sleeping Tablets, e.g., Zopiclone (Yes)**	7.330	0.203	0.342	156.989
**Have you received mental health counseling in the past year? (Yes)**	0.073	0.033	0.007	0.814
**Would you like to receive mental health counseling?**				
No		0.015		
Yes	20.762	0.004	2.606	165.401
Unsure/Undecided	16.674	0.010	1.985	140.063
**ACE Score**				
0		0.034		
1	0.488	0.506	0.059	4.042
2	1.039	0.968	0.161	6.720
3	0.412	0.360	0.061	2.758
Four or more	6.243	0.013	1.461	26.673
Constant		0.075		

^C^.I: Confidence Interval.

Table 4 and Table in S2 Table indicates that 16 variables had statistically significant associations (*p* ≤ 0.05) or near significant associations (0.1 ≥ *p* > 0.05) with low resilience. Respondents who were aged 26 years or less, of other gender or other ethnicity, had less than high school education, were unemployed, separated/divorced/widowed, living with family and friends, shown likely low resilience compared to other participants in their respective categories. Similarly, respondents with a mental health history of depression, anxiety disorder, PD, OCD, PTSD, those with no mental health history, those on no medication or antidepressants, sleeping tablets, those who would like to receive mental health counseling, or have received mental health counseling and those with 3 ACEs showed low resilience compared with other participants in their respective categories.

**Table 4:** Chi-square test between demographic, mental health characteristics, and low resilience.

Variables	Normal-to-High Resilience n (%) N = 102	Low Resilience n (%) N = 109	P Value
**Socio-demographic characteristics**			
**Age (Years)**			< 0.001
≤26	41 (36.3%)	72 (63.7%)	
>26	61 (62.2%)	37 (37.8%)	
**Educational level**			< 0.001
Less than High School	6 (24.0%)	19 (76.0%)	
High school	14 (30.4%)	32 (69.6%)	
Post-secondary education	82 (58.6%)	58 (41.4%)	
**Relationship status**			*0.03
In a relationship (married, common law, partnered)	56(58.3%)	40 (41.7%)	
Single	43 (40.6%)	63 (59.4%)	
Separated/Divorced/Widowed	3 (33.3%)	6 (66.7%)	
**Employment status**			0.02
Employed	63 (58.9%)	44 (41.1%)	
Unemployed	8 (32.0%)	17 (68.0%)	
Student	18 (39.1%)	28 (60.9%)	
Student and employed	13 (39.4%)	20 (60.6%)	
**Housing status** Own home	44 (69.8%)	19 (30.2%)	< 0.001
Rented accommodation	24 (42.1%)	33 (57.9%)	
Live with family or friend	34 (37.4%)	57 (62.6%)	
**MH history**			
Depression			0.003
No	73 (56.6%)	56 (43.4%)	
Yes	29 (35.4%)	53 (64.6%)	
GAD			< 0.001
No	73 (58.9%)	51 (41.1%)	
Yes	29 (33.3%)	58 (66.7%)	
OCD			0.01
No	98 (51.3%)	93 (48.7%)	
Yes	4 (20.0%)	16 (80.0%)	
PD			*0.01
No	102 (50.0%)	102 (50.0%)	
Yes	0 (0.0%)	7 (100.0%)	
PTSD			0.09
No	91 (50.8%)	88 (49.2%)	
Yes	11 (34.4%)	21 (65.6%)	
No MH history			0.01
No	54 (58.7%)	38 (41.3%)	
Yes	48 (40.3%)	71 (59.7%)	
**Medication Hx**			
Antidepressants			0.07
No	77 (52.7%)	69 (47.3%)	
Yes	25 (38.5%)	40 (61.5%)	
Sleeping tablets			0.002
No	101 (51.0%)	97 (49.0%)	
Yes	1 (7.7%)	12 (92.3%)	
No medications			0.05
No	30 (39.5%)	46 (60.5%)	
Yes	72 (53.3%)	63 (46.7%)	
**Have you received MH counseling?**			0.01
No	59 (57.8%)	43 (42.2%)	
Yes	43 (39.4%)	66 (60.6%)	
**Would you like to receive MH counseling**			0.002
No	25 (83.3%)	5 (16.7%)	
Yes	18 (43.9%)	23 (56.1%)	
Unsure/Undecided	22 (51.2%)	21 (48.8%)	

*Fishers Exact Value.

Table 5 below shows the logistic regression model for BRS. Overall, 14 predictors that showed significant association or a trend towards significant association (*p* ≤ 0.05) or near significance (0.1 ≥ *p* > 0.05) with low resilience on the Chi-Square/ Fisher’s exact test were included in a binary logistic regression model. Two highly correlated (r ≥ 0.7) variables were excluded from the regression analysis. The excluded variables included “housing status,” which was highly correlated with “age,” and “have never received mental health diagnosis,” which was highly correlated with “history of anxiety disorder.” In each case, the rationale for excluding the former instead of the latter from the logistic regression model was that the latter variables were perceived to be more relevant to the outcome of interest. The logistic regression model was statistically significant; *Χ*^2^ (df = 19; *n* = 351) = 63.46, *p* ≤ 0.001, indicating that the model could distinguish between study participants with the presence and absence of low resilience. The model explained 42.7% (Cox and Snell R^2^) to 57.3% (Nagelkerke R^2^) of the variance and correctly classified 81.6% of cases. Education as a variable did not significantly contribute to the model. However, participants who completed post-secondary education were 17.5 times more likely to have low resilience compared to participants who had less than high school education (OR = 17.5; 95% CI: 1.56–200.00). Other variables*, “Have you received mental health counseling in the past year?” and* “*Would like to receive mental health counseling,”* independently predicted the presence of low resilience in the respondents. When all other variables are controlled in the regression model, participants who received mental health counseling in the past year were 15.15 times more likely to experience low resilience than those who did not (OR = 15.15; 95% CI: 1.46–166.67).

**Table 5:** Output of the logistic regression model illustrating predictors of low resilience.

	Exp(B) Odd Ratio (OR)	Sig.	95% C.I.for EXP(B)	
			Lower	Upper
**Age (years)** **≤ 26**	0.419	0.271	0.089	1.970
**Education**				
Less than high school		0.067		
High school	0.140	0.090	0.014	1.355
Post-secondary education	0.057	**0.020**	0.005	0.639
**Relationship status**				
In a relationship (married, common law, partnered)		0.732		
Single	0.586	0.430	0.155	2.208
Separated/Divorced/Widowed	0.000	0.999	0.000	
**Employment status**				
Employed				
Unemployed	4.227	0.106	0.735	24.318
Student	3.981	0.213	0.453	34.994
Student and employed	1.665	0.592	0.258	10.735
**Depression Disorder diagnosis? (Yes)**	4.313	0.077	0.853	21.809
**Anxiety disorder mental health diagnosis (Yes)**	1.486	0.649	0.270	8.178
**Obsessive-compulsive Disorder diagnosis? (Yes)**	2.672	0.346	0.346	20.627
**Have you received a Personality Disorder Diagnosis (Yes)**	0.000	1.000	0.000	
**ADHD diagnosis (Yes)**	2076744844	0.999	0.000	
**Have you received a PTSD or trauma-related disorder diagnosis? (Yes)**	0.118	0.096	0.009	1.461
**Medication**				
**On Antidepressants, e.g., Prozac (Yes)**	0.310	0.234	0.045	2.134
**On Sleeping Tablets, e.g., Zopiclone (Yes)**	8865656303	0.999	0.000	
**Have you received mental health counseling in the past year? (Yes)**	0.066	**0.023**	0.006	0.683
**Would you like to receive mental health counseling?**				
No		**0.007**		
Yes	29.417	**0.002**	3.310	261.445
Unsure/Undecided	30.617	**0.003**	3.320	282.379
Constant	0.451	0.611		

Similarly, those who would like to receive mental health counseling were 29.42 times more likely to experience low resilience than those who would not (OR = 29.42; 95% CI: 3.31–261.445). Additionally, those who were not sure if they would like to receive mental health counseling were 30.62 times more likely to experience low resilience than those who did not want to receive mental health counseling (OR = 30.62.62; 95% CI: 3.32–282.379).

## Discussion

### Prevalence and predictors of probable PTSD

PTSD has been reported to be prevalent among adolescents and young adults and is associated with increased experience of traumatic events in their lifetime [1, 2]. The presence of trauma and other mental health issues have also been reported to heighten the possibility of probable PTSD in the general population [8]. The COVID-19 pandemic has been traumatic for the general public, but adolescents and young adults appear to have been disproportionately impacted [34]. In this study, the prevalence of probable PTSD among the younger adult subscribers of MoreGoodDays was 58.6% compared to 30.9% for those older than 26 years. Comparatively, the prevalence of probable PTSD in young adults is much higher than in the region of Alberta, impacted by wildfires, which reported a prevalence of 36.8% [28]. The COVID-19 pandemic had an adverse effect on adolescents’ and young adults’ mental health and is evident in the significant increase in the prevalence of probable PTSD in our study. The high prevalence of low resilience and PTSD symptoms among young adults may be explained through psychological mechanisms and their relationships. Their developmental stage is often associated with heightened emotional sensitivity, which may magnify their responses to traumatic events [35]. This can make young adults more susceptible to stress and trauma, potentially leading to the increased prevalence of PTSD symptoms and low resilience, as observed in our study. During the COVID-19 pandemic, particularly during the lockdown period, young adults may have faced increased social isolation, lack of supportive relationships, and decreased peer-to-peer interactions, all of which may have been traumatic for even individuals who were not necessarily infected by the COVID-19 virus.

Other studies have reported a relatively lower prevalence of probable PTSD. For example, a very low prevalence of 2.7% probable PTSD was reported in a study one month after the COVID-19 outbreak in China. This low prevalence of probable PTSD may be attributed to the time of the study. MoreGoodDays was introduced after the quarantine and during increased uncertainty and awareness about the pandemic. One study [3] also reported a prevalence of 29.4% probable PTSD, which was still lower than what has been recorded for participants in our study. Another study among high school students after a wildfire reported a 37% prevalence of probable PTSD [36].

In terms of predictors of probable PTSD, this study has established that demographic variables, including age, education, employment, and relationship status, and clinical variables, such as history of depression, anxiety, PD, and ADHD, did not independently predict probable PTSD in subscribers of MoreGoodDays. One study, reported that adolescents with PTSD had higher anxiety and depression symptoms, and 47% of youth who had probable PTSD also had a depression diagnosis [7]. However, another study among the general public also reported no significant associations between probable PTSD and age groups, relationship status, employment, and educational status [37]. On the contrary, a history of depression diagnosis was a predictor of probable PTSD, with respondents being four times more likely to have probable PTSD than those who did not have a prior diagnosis of depression [38].

The present study observed that both previous receipts of mental health counseling and the desire to receive the same were predictors of PTSD. Respondents who have had mental health counseling in the past twelve months were 13.7 times more likely to experience probable PTSD than participants who had not. Again, those who desired mental health counseling were 20.8 times more likely to experience PTSD symptoms than respondents who did not. A recent study also endorsed willingness to receive mental health counseling as a predictor of PTSD [38], and the desire to receive mental health counseling has been proposed as a better predictor of mental health problems following traumatic events [39]. It may be possible that individuals experiencing more severe symptoms might be more likely to seek or express a desire for counselling, rather than counselling being a contributing factor to PTSD symptoms.

The COVID-19 pandemic caused an increase in psychological issues, leading to a rise in the desire for mental health counseling or psychological knowledge and interventions among college students [40, 41]. The subscribers of MoreGoodDays who had received mental health counseling in the past may have had past or ongoing mental health issues, which increases their risk for the development of PTSD. The current study also observed that participants who had four or more Adverse Childhood Experience (ACE) scores were 6.2 times more likely to experience PTSD symptoms than those who had zero scores. This is supported by a study among health science students, which noted significantly worse mental health outcomes among participants with four or more ACEs [42].

### Prevalence and predictors of low resilience

The prevalence of low resilience among the subscribers of MoreGoodDays 26 years and younger was 63.7%, and 37.8% for participants older than 26 years. The high prevalence of low resilience among participants 26 years and younger in this study is consistent with a previous study that reported that young individuals are more prone to low resilience than older individuals [43]. A probable explanation for the high incidence of low resilience in this study is that adolescents and young adults had poor ability to cope with the trauma of the pandemic. Generally, resilience requires effective adaptation to stressful, adverse, or traumatic events and avoidance of stress-induced mental issues such as PTSD [44]. Experience of traumatic events has often been associated with an increased likelihood of low resilience and possible PTSD. The COVID-19 pandemic may have challenged the resilience among the subscribers of MoreGoodDays. Additionally, there was a worldwide decline in resilience during the COVID-19 pandemic, with a 35.0% prevalence of low resilience among the general population and 1 in 4 people experiencing low resilience [45]. This reflects the prevalence of low resilience reported in our study among young adults.

In this study, socio-demographic variables, like age, employment, and relationship status, and clinical variables, such as the history of anxiety, depression, PD, OCD, ADHD, PTSD, and ACE scores, did not independently predict likely low resilience. Our findings contradict another study, which reported that age was independently associated with low resilience [46]. Another study also reported a significant association between age and resilience, suggesting that older people were more resilient [43]. Furthermore, prior experience has been suggested to improve resilience with lived experience, and hence, older people, particularly those whom COVID-19 less threatened, had higher resilience [47]. One study also reported that a history of anxiety disorder and depression were significant predictors of low resilience among the general population [43], which contradicts our study findings. Our results also contradict another study that reported an association between high resilience and a lower prevalence of mental health problems such as depression, anxiety, and PTSD [28].

Education as a variable did not significantly contribute to the regression model. However, participants who completed post-secondary education were 17.5 times more likely to have low resilience compared to participants who had only a high school education or less. Contrary results were reported in another study, which suggested that high resilience was more prevalent in individuals with higher education [47]. One study among college students reported that those negatively affected by stress also performed poorly and had low resilience [48]. Research indicates that individuals may be less resilient when exposed to multiple disasters in addition to the COVID-19 pandemic, and resilient behaviors have been suggested to have a predictive effect on mental health problems [49, 50]. Therefore, a plausible explanation for the results of the current study may be that the COVID-19 pandemic, coupled with the increased stress associated with post-secondary education, might have led to a decline in resilience.

The receipt and desire to receive mental health counseling independently predicted low resilience in the current study. A pattern of the increased likelihood of low resilience is determined approximately 15 times, 29 times, and 31 times, respectively**,** for those who have received, desire to receive, and are unsure of receiving mental health counseling. A probable explanation for this trend is that mental health counseling may depict the possibility of psychological issues. Therefore, receipt of counseling or desire to receive or contemplation of the same may be an indication that participants had an increased psychological burden, leading to lower resilience. The current study did not explore the duration or kind of counseling MoreGoodDays subscribers had previously received. Future research may explore this further to determine the kind and exact duration of counseling and its effects on resilience.

### Limitations

It is worth mentioning that though the scales used to assess mental health outcomes are validated and standardized proxy scales, they are not diagnostic. The actual prevalence of probable PTSD in this cohort may, therefore, differ from what has been recorded using the screening tool. The current study data were collected via a self-reported online survey, so responses cannot be independently verified, which may possibly affect the interpretation and generalizability of outcome. Again, the completion of the survey by MoreGoodDays subscribers were voluntary, which may have introduced potential selection bias, as such, the findings may not be generalizable to the broader youth population.

In addition, more than seventy percent of our study respondents were female, which is not representative of the demographics of young adults in Alberta or Canada. Thus, the study outcome may not be generalizable to Alberta’s adolescent and young adult population or reflect other ethnic diverse group due to their limited representation. Also, given the lower sample size of 343 recorded for this study, the margin of error for prevalence estimates was higher at 4% at a 95% confidence level rather than the projected 3% estimated using a projected sample size of 529. Additionally, the wide range of confidence intervals reported in our study, may be possibly due to small subgroup sizes or low statistical power. Furthermore, given the cross-sectional nature of this study, causality cannot be inferred between counselling and symptom severity. At best, this study confirms an association between these two variables. Again, it is possible that individuals experiencing psychological trauma might have been more likely to subscribe to the MoreGoodDays program and completed the survey, thus inflating the prevalence of probable PTSD.

Notwithstanding these limitations, the current study gives valuable data on the prevalence of probable PTSD and low resilience and their predictors among subscribers of an e-mental health intervention designed for adolescents and young adults.

### Conclusions and implication for policy and practice

The current study results give insight into the prevalence and predictors of resilience and probable PTSD among subscribers of MoreGoodDays, an e-mental health program co-designed for Alberta’s adolescents and young adults. MoreGoodDays subscribers, especially adolescents and young adults, were disproportionally more likely to report probable PTSD symptoms and low resilience, reflecting the devastating effect of the COVID-19 pandemic on this cohort of subscribers. Increased ACE has been linked to low resilience, which may also lead to a rise in mental health issues.

Nonetheless, it is worth mentioning that past traumatic experiences prior to the onset of the pandemic may have been a contributing factor. Additionally, though participants cultural variation in relation to trauma may influence their resilience, this was not a variable of interest in this study. Future research may explore this variable and its relationship with resilience and probable PTSD. Intervention strategies promoting resilience are timely and appropriate and may inadvertently reduce the risk and incidence of probable PTSD among this cohort. MoreGoodDays may be adapted, personalized and the content of the messages tailored for users with high ACE scores to help improve their resilience. Future studies should also aim to conduct longitudinal follow-up or randomized controlled trials to assess the effectiveness of the MoreGoodDays intervention. It is recommended that policymakers in the educational field review the curriculum and incorporate measures aimed at building and increasing resilience, as this will be significant in reducing or preventing mental health problems, including probable PTSD, among this group. According to a systematic review, this approach also positively impacts young people’s capability to manage everyday stressors [51]. In addition, innovative mental health interventions such as supportive mobile text messaging technology [17, 21] may be adapted to assist the psychological health of adolescents, young adults, and the general public.

The study provides vital information on how mental health resources can be allocated to support young adults who may be vulnerable effectively and this may significantly impact theoretical frameworks and policy-making and contribute to improved mental health outcomes. The findings of this study also highlight the need for scalable, geographic location-independent, trauma-informed SMS-based interventions, such as the MoreGoodDays program, as potential strategies to support youth mental well-being. Adolescents and young adults are generally more engaged in a digital intervention thus; our findings offer context-specific insights into addressing mental health needs in this cohort.

Furthermore, mental health literacy should be integrated in schools, and educators should be trained to recognize early signs of mental distress. Finally, policymakers and government agencies are also encouraged to give the mental health of young adults and youth more prominence on their political agenda.

### Informed consent statement

This research was conducted in accordance with the Declaration of Helsinki, and ethics approval was granted by the University of Alberta Health Research Ethics Committee (Pro00106957). Informed consent was obtained from all subjects.

## Supporting information

S1 Table Chi-square test of association between demographic, mental health characteristics, and Probable PTSD. (DOCX)

S2 Table Chi-square test between demographic, mental health characteristics, and Low Resilience. (DOCX)
