# Supplementary material for: Prevalence and predictors of PTSD and low resilience symptoms among subscribers of the MoreGoodDays supportive text messaging program: A cross-sectional study
Source: PLoS One. 2026 Jan 9;21(1):e0339662. doi: 10.1371/journal.pone.0339662 (PMC12788678; doi:10.1371/journal.pone.0339662)
Supplement: S1 Table — (DOCX) [file pone.0339662.s001.docx]

**S1 Table: Chi-square test of association between demographic, mental health characteristics, and Probable PTSD.**

| **Variables** | **Unlikely PTSD**  **n (%)**  **N= 113** | **Probable PTSD**  **n (%)**  **N= 95** | **P value** |
| --- | --- | --- | --- |
| **Socio-demographic characteristics** | |  |  |
| **Gender**  Male  Female  Other | 17 (51.5%)  91 (55.5%)  5 (45.5%) | 16 (48.5%)  73 (44.5%)  6 (54.5%) | 0.78 |
| **Ethnicity**  White  Aboriginal  Asian  African Descendants  Other | 88 (56.4%)  7 (43.8%)  11 (52.4%)  2 (50.0%)  5 (45.5%) | 68 (43.6%)  9 (56.3%)  10 (47.6%)  2 (50.0%)  6 (54.5%) | *0.84 |
| **MH history** | |  |  |
| BD  No  Yes | 110 (55.0%)  3 (37.5%) | 90 (45.0%)  5 (62.5%) | *0.47 |
| Eating disorder  No  Yes | 105 (55.3%)  8 (44.4%) | 85 (44.7%)  10 (55.6%) | 0.46 |
| OCD  No  Yes | 104 (55.3%)  9 (45.0%) | 84 (44.7%)  11 (55.0%) | 0.48 |
| SUD  No  Yes | 112 (55.2%)  1 (20.0%) | 91 (44.8%)  4 (80.0%) | *0.18 |
| Schizophrenia  No  Yes | 111 (53.9%)  2 (100.0%) | 95 (46.1%)  0 (0.0%) | *0.50 |
| **Medication Hx** |  |  |  |
| Antipsychotic  No  Yes | 109 (54.5%)  4 (50.0%) | 91 (45.5%)  4 (50.0%) | 1.00 |
| Benzodiazepines  No  Yes | 110 (55.0%)  3 (37.5%) | 90 (45.0%)  5 (62.5%) | *0.47 |
| Mood stabilizers  No  Yes | 110 (55.0%)  3 (37.5%) | 90 (45.0%)  5 (62.5%) | *0.47 |
| Stimulants  No  Yes | 110 (55.3%)  3 (33.3%) | 89 (44.7%)  6 (66.7%) | *0.31 |
