# Supplementary material for: Prevalence and predictors of PTSD and low resilience symptoms among subscribers of the MoreGoodDays supportive text messaging program: A cross-sectional study
Source: PLoS One. 2026 Jan 9;21(1):e0339662. doi: 10.1371/journal.pone.0339662 (PMC12788678; doi:10.1371/journal.pone.0339662)
Supplement: S2 Table — (DOCX) [file pone.0339662.s002.docx]

**S2 Table: Chi-square test between demographic, mental health characteristics, and Low Resilience.**

| **Variables** | **Normal-to-High Resilience**  **n (%)**  **N= 102** | **Low Resilience**  **n (%)**  **N= 109** | **P Value** |
| --- | --- | --- | --- |
| **Socio-demographic characteristics** | |  |  |
| **Gender**  Male  Female  Other | 20 (58.8%)  78 (47.0%)  4 (36.4%) | 14 (41.2%)  88 (53.0%)  7 (63.6%) | 0.34 |
| **Ethnicity**  White  Aboriginal  Asian  Other | 80 (48.8%)  9 (52.9%)  11 (52.4%)  2 (22.2%) | 84 (51.2%)  8 (47.1%)  10 (47.6%)  7 (77.8%) | *0.45 |
| BD  No  Yes | 99 (49.0%)  3 (33.3%) | 103 (51.0%)  6 (66.7%) | *0.28 |
| Eating disorder  No  Yes | 96 (50.0%)  6 (31.6%) | 96 (50.0%)  13 (68.4%) | 1.00 |
| SUD  No  Yes | 99 (48.3%)  3 (50.0%) | 106 (51.7%)  3 (50.0%) | 1.00 |
| Schizophrenia  No  Yes | 100 (47.8%)  2 (100.0%) | 109 (52.2%)  0 (0.0%) | *0.23 |
| ADHD  No  Yes | 99 (49.7%)  3 (25.0%) | 100 (50.3%)  9 (75.0%) | 1.00 |
| **Medication Hx** |  |  |  |
| Antipsychotic  No  Yes | 99 (49.0%)  3 (33.3%) | 103 (51.0%)  6 (66.7%) | *0.50 |
| Benzodiazepines  No  Yes | 98 (48.3%)  4 (50.0%) | 105 (51.7%)  4 (50.0%) | *1.00 |
| Mood stabilizers  No  Yes | 98 (48.5%)  4 (44.4%) | 104 (51.5%)  5 (55.6%) | *1.00 |
| Stimulants  No  Yes | 98 (48.5%)  4 (44.4%) | 104 (51.5%)  5 (55.6%) | *1.00 |
| **ACE score**  **0**  **1**  **2**  **3**  **Four or more** | 31 (58.5%)  14 (45.2%)  16 (50.0%)  7 (31.8%)  30 (44.1%) | 22 (41.5%)  17 (54.8%)  16 (50.0%)  15 (68.2%)  38 (55.9%) | 0.27 |
